# Birthweight measurement processes and perceived value: qualitative research in one EN-BIRTH study hospital in Tanzania

**DOI:** 10.1186/s12884-020-03356-2

**Published:** 2021-03-26

**Authors:** Miriam E. Gladstone, Nahya Salim, Karama Ogillo, Donat Shamba, Georgia R. Gore-Langton, Louise T. Day, Hannah Blencowe, Joy E. Lawn, EN-BIRTH Study Group, Nahya Salim, Donat Shamba, Josephine Shabani, Kizito Shirima, Menna Narcis Tarimo, Godfrey Mbaruku, Honorati Masanja, Louise T. Day, Harriet Ruysen, Kimberly Peven, Vladimir S. Gordeev, Georgia R. Gore-Langton, Dorothy Boggs, Stefanie Kong, Angela Baschieri, Simon Cousens, Joy E. Lawn

**Affiliations:** 1 Centre for Maternal, Adolescent, Reproductive, & Child Health (MARCH), London School of Hygiene and Tropical Medicine (LSHTM), London, UK; 2 Department of Health Systems, Impact Evaluation and Policy, Ifakara Health Institute (IHI), Dar es Salaam, Tanzania; 3 Department of Paediatrics and Child Health, Muhimbili University of Health and Allied Sciences, Dar es Salaam, Tanzania

**Keywords:** Birthweight, Birth, Hospital, Neonatal, Maternal, Coverage, Weighing scale

## Abstract

**Background:**

Globally an estimated 20.5 million liveborn babies are low birthweight (LBW) each year, weighing less than 2500 g. LBW babies have increased risk of mortality even beyond the neonatal period, with an ongoing risk of stunting and non-communicable diseases. LBW is a priority global health indicator. Now almost 80% of births are in facilities, yet birthweight data are lacking in most high-mortality burden countries and are of poor quality, notably with heaping especially on values ending in 00. We aimed to undertake qualitative research in a regional hospital in Dar es Salaam, Tanzania, observing birthweight weighing scales, exploring barriers and enablers to weighing at birth as well as perceived value of birthweight data to health workers, women and stakeholders.

**Methods:**

Observations were undertaken on type of birthweight scale availability in hospital wards. In-depth semi-structured interviews (*n* = 21) were conducted with three groups: women in postnatal and kangaroo mother care wards, health workers involved in birthweight measurement and recording, and stakeholders involved in data aggregation in Temeke Hospital, Tanzania, a site in the EN-BIRTH study. An inductive thematic analysis was undertaken of translated interview transcripts.

**Results:**

Of five wards that were expected to have scales, three had functional scales, and only one of the functional scales was digital. The labour ward weighed the most newborns using an analogue scale that was not consistently zeroed. Hospital birthweight data were aggregated monthly for reporting into the health management information system. Birthweight measurement was highly valued by all respondents, notably families and healthcare workers, and local use of data was considered an enabler. Perceived barriers to high quality birthweight data included: gaps in availability of precise weighing devices, adequate health workers and imprecise measurement practices.

**Conclusion:**

Birthweight measurement is valued by families and health workers. There are opportunities to close the gap between the percentage of babies born in facilities and the percentage accurately weighed at birth by providing accurate scales, improving skills training and increasing local use of data. More accurate birthweight data are vitally important for all babies and specifically to track progress in preventing and improving immediate and long-term care for low birthweight children.

**Supplementary Information:**

The online version contains supplementary material available at 10.1186/s12884-020-03356-2.

## Key findings

**Table:** 

**What is known and what is new about this study?**
• Birthweight data are essential for tracking progress towards the World Health Organization’s global nutrition targets regarding low birthweight by 2025, and as a predictor of neonatal deaths and long-term health outcomes. However, birthweight data from routine facility data systems are lacking in most of sub-Saharan Africa and South Asia, despite most births now being in facilities. • Our study is one of the first to explore perceptions of birthweight measurement. In a regional hospital in Tanzania, we sought to understand factors contributing to the birthweight data gap by documenting equipment availability and assessing attitudes towards measurement by women who had recently given birth, health workers and public health stakeholders.
**Observation of weighing scales**
• High quality birthweight information requires functioning, calibrated, accurate weighing scales. The labour and delivery ward used an analogue weighing scale observed to be not calibrated to zero. Of newborn weighing scales in four other hospital wards: two were digital, two were analogue and only half were functioning.
**In-depth semi-structured interviews – what did we find and what does it mean?**
• *Collection:* Barriers to high quality birthweight measurement included lack of precise equipment, no standardised technical weighing protocols and health worker shortage. • *Perceived value:* Women and healthcare workers highly value birthweight measurement and perceive its use to inform appropriate treatment as needed, including medication dosage, and to monitor growth. This perception created a positive view for high quality facility birthweight measurement. • *Utility:* Perceived poor data quality was reported to limit effective usage of birthweight reported though the Health Management Information Systems (HMIS).
**What next and research gaps?**
• Using facility birthweight data is increasingly important for tracking national and global LBW rates. Opportunities exist to close the data quality gap for facility births, notably through improvements in equipment, training and human resources. Implementation research is needed to understand how digital scales and improved weighing protocols and practices can strengthen the quality of birthweight data, e.g. reducing heaping. Further research is also required to evaluate data flow in routine HMIS and if improved quantity and quality of data increases confidence in use of birthweight data.

## Background

Low birthweight (LBW) is defined as a birthweight of less than 2500 grammes (g), and affected an estimated 20.5 million newborns globally in 2015 [1]. Over 80% of the world’s 2.5 million annual newborn deaths are LBW [2]. LBW can be a result of preterm birth, intrauterine growth restriction or a combination of both. Compared to normal birthweight infants, LBW neonates experience increased morbidity, including acute neonatal complications (e.g. preterm respiratory distress, hypothermia and hypoglycaemia) as well as childhood stunting and a risk of adult-onset chronic conditions (e.g. cardiovascular disease) [3–6]. Accurate birthweight is important at the individual level to enable provision of life-saving interventions: extra warmth, feeding support and increased focus on detection and treatment of complications [7, 8]. Calculating appropriate drug doses, fluids and milk volumes also require a correct birthweight. Birthweight measurement is an important baseline from which to measure growth for all newborns [9].

At population level LBW is also important, especially for tracking national targets. The Sustainable Development Goals have the first global target to end preventable newborn deaths by 2030. Multiple countries are implementing programmes to reach national targets based on the *Every Newborn* Action Plan (ENAP) [10]. One of five *Every Newborn* strategic objectives is to improve measurement, including for birthweight, as outlined in the linked measurement improvement roadmap [11]. LBW rate is a priority target in the global nutrition targets committed to decreasing global LBW prevalence by 30% before 2025 [4]. Hence policy makers need accurate LBW data to assess progress and target investments [12].

Accurate birthweight measurement requires newborns to be weighed within an hour of birth using a well-calibrated scale measuring in 10 g increments [3, 13]. To prevent cross-infection, a thin clean cloth or paper should be placed on the scale. The device is then zeroed, the newborn placed on the scale naked, and the weight allowed to stabilise before being captured and recorded [9, 14]. Although true birthweights are normally distributed, heaping of birthweight measurements is common in low- and middle-income countries (LMIC) [15–19]. Birthweight heaping at 2500 g may result in LBW infants being misclassified as normal birthweight. In addition, birthweight rounding also occurs due to the phenomena of “digit bias”, for numbers ending in 0 or 5 [16, 20, 21].

Facility births now account for around 80% of births worldwide [22], so facility measured birthweight is an increasingly important data source to track LBW prevalence through the Health Management Information System (HMIS) [1, 23]. However, LBW data availability remains a challenge especially in the highest mortality burden settings in sub-Saharan Africa and south Asia [1, 22, 23]. Birthweight data from both household surveys and facilities have been shown to be of mixed quality with high degrees of missing data and heaping [15, 16, 20, 24, 25].

In the Tanzania Demographic and Health Surveys (DHS) 2016 report, birthweight data were reported for 63.5% of live births [26–28]. For homebirths, timely birthweight measurement is usually not possible and survey questions to the mother may rely on her perception of birthweight [15, 28–30]. In Tanzania, facility labour ward birthweight data are aggregated for entry into HMIS, specifically District Health Information Software 2 (DHIS-2). Thus HMIS now has the potential to provide regular birthweight data for the 62.8% of births that now take place in facilities in Tanzania, alongside birthweight data from population-based surveys [28]. However, concerns regarding the quality of facility birthweight data could limit the usefulness of this data source.

We identified no previous published research regarding perceptions of women, healthcare providers, or other stakeholders regarding birthweight measurement in facilities in Tanzania, or elsewhere in sub-Saharan Africa. Prior research on value of birthweight has been in settings with high homebirth rates. In rural India, birthweight was not considered as an important measurement or determinant of newborn health by women, their families or health stakeholders [31]. Similarly, in rural Bangladesh participants did not prioritise birthweight measurement or recognise its importance for monitoring newborn health [32].

This study is nested within one hospital of the five sites in the *Every Newborn*-Birth Indicators Research Tracking in Hospitals (EN-BIRTH) study [11, 33, 34].

## Objectives

This paper is part of a supplement based on the EN-BIRTH multi-country validation study, *‘Informing measurement of coverage and quality of maternal and newborn care’*, and aims to identify opportunities to improve quality of facility birthweight data through the following objectives:

1. **Identify AVAILABLE WEIGHING SCALES** in Temeke hospital.
2. **Explore BARRIERS AND ENABLERS to accurate birthweight measurement** with perceived value and use of birthweight data by women, health workers and public health/other hospital stakeholders.

## Methods

### Setting

Temeke Hospital is a 294 bed regional referral hospital serving a district population of > 760,000 located in Dar es Salaam, Tanzania (TZ) [35]. The hospital was selected as one of two sites in Tanzania for the wider EN-BIRTH validation study as public hospitals providing the selected interventions for validity assessment of indicator measures. This birthweight study took place in only one of these two hospitals to enable the level of detail needed [33]. Birthweight is recorded in the national standardised HMIS Book 12 Register on the labour ward. Postnatal mothers and babies are transferred to three wards: ‘Postnatal ward A’ after caesarean section, ‘Postnatal ward B’ after vaginal births or the kangaroo mother care (KMC) ward. Temeke Hospital policy admits stable babies weighing < 2500 g in the KMC ward, unlike the WHO KMC guidelines, which include babies ≤ 2000 g [36]. Unstable newborns are transferred to a neonatal ward. 14 nurses/midwives in the labour ward and 9 nurses/midwives in the KMC ward are involved in measuring birthweight.

### Study design

This study triangulated the identification and observation of the availability, type, and appearance of existing weighing scales at Temeke Hospital (Objective 1) within a predominantly qualitative approach (Objective 2).

### Objective 1: Identify available weighing scales

Observation was made once by two research assistants on the availability, type and appearance of newborn weighing scales at Temeke Hospital in all wards caring for newborns and mothers: Labour ward, Postnatal A and Postnatal B, KMC and Maternal Intensive Care Unit (ICU). A digital photo was taken of each study scale.

### Objective 2: Perceptions of birthweight measurement, documentation, significance and use

Women enrolled in the EN-BIRTH study at Temeke Hospital after birth of a live born baby or admitted to the KMC ward were recruited after the EN-BIRTH exit interview survey. Temeke Hospital nurses/midwives routinely involved in weighing newborn babies were recruited by snowball sampling after an initial interview with a KMC ward nurse. Once snowball sampling was exhausted, purposive sampling was used to recruit nurses/midwives from underrepresented wards. Women and nurses/midwives were recruited until saturation when the interviews generated no new information. KMC ward nurses identified a doctor and hospital administrator who were involved in birthweight data aggregation and use. Departments of health at the municipal and national level that use birthweight data were identified and recruitment continued until each department had representation. Written informed consent was taken in the participants’ preferred language (English or Swahili) prior to interview. All participants were able to provide written consent.

Following review of the literature, interview guides on knowledge, attitudes and practices surrounding birthweight measurement were drafted, translated into Swahili and revised for local acceptability (Additional files 1 and 2). The guides were piloted with women who had given birth and nurses/midwives at Temeke Hospital who matched the study inclusion criteria and revised accordingly. Guides used for stakeholders were not piloted because of the limited number of stakeholders. However, due to their semi-structured nature, the interviews were flexible and able to capture varied responses. A Tanzanian female research assistant and the first author recruited participants and conducted the in-depth semi-structured interviews in a private room within Temeke Hospital or in the stakeholder’s office. Interviews were conducted in English or Swahili at the respondent's preference and when in Swahili were translated verbatim in real-time into English by the research assistant. Interviews lasted approximately 30 min in duration and no repeat interviews were conducted. Interviews were recorded, transcribed, translated verbatim, anonymised and stored on a secure server. An inductive thematic analysis was undertaken using NVivo 10 for data management [37–39]. The first author read the transcripts for general impression then generated initial codes inductively. To improve the trustworthiness of the results, multiple researchers commented on and contributed to the grouping of codes with similar concepts into themes and sub-themes to create a conceptual framework and interpret findings. Disagreements in interpretation was resolved by consensus. Themes were compared across different groups of participants to assess differences and similarities in views, results were triangulated among participants and representative quotations were selected. Coding themes are described in Additional file 3.

Credibility of findings was attained through a prolonged research engagement with the Temeke site and through triangulation of data collection methods, of responses between populations and of interpretation of results between researchers. Detailed records were maintained throughout data collection and analysis to strengthen dependability of results. Some generalisability of the results was supported through purposive sampling of the research site and of respondents. Results are reported in accordance with the consolidated criteria for reporting qualitative research (COREQ) checklist (Additional file 4) [40].

## Results

### Objective 1: Observation of weighing scales

**Fig. 1:**
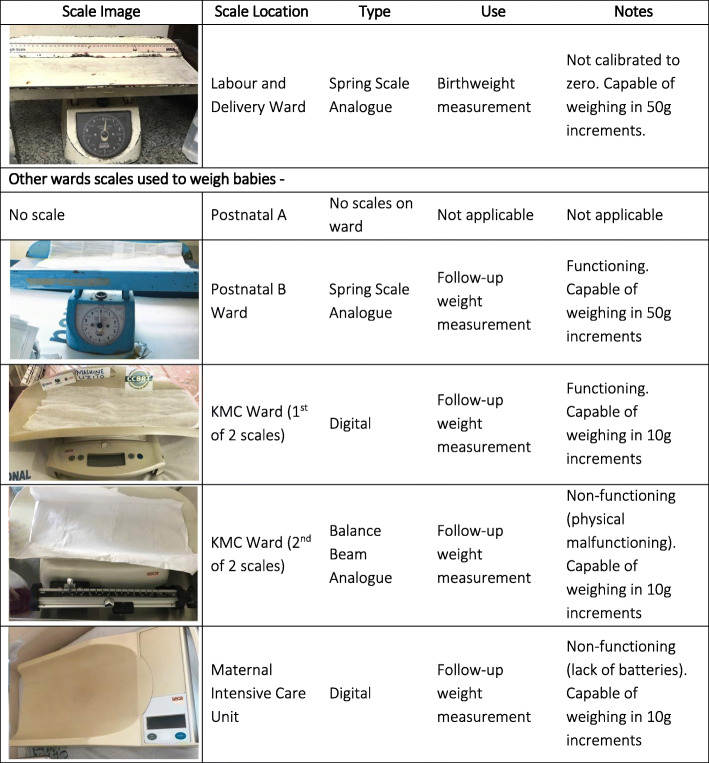
Characteristics of scales observed at Temeke Hospital, EN-BIRTH study. Assessments undertaken in labour and delivery ward, and places of newborn care

Weighing scales were found on four of the five inpatient wards caring for newborns, of which only three were functioning. The functioning analogue scale in the labour ward, usually used for birthweight measurement, was capable of weighing in 50 g increments, but was noted not to be zeroed with the paper laid on it. In the KMC ward one digital scale was functioning and capable of weighing in 10 g increments and a second analogue scale was non-functioning. In the maternal ICU the digital scale had no batteries (Fig. 1). No scale was found in postnatal ward A and the functioning analogue scale in postnatal ward B was capable of weighing in 50 g increments (Fig. 1).

### Objective 2: In-depth semi-structured interviews

21 participants were interviewed and no one approached refused to participate. The first group of participants were 8 women (four with LBW babies admitted on KMC ward and four with babies of normal weight discharged from postnatal ward B). The second group were 10 health workers (nine nurses/midwives and one doctor) who had a mean working experience of 5.3 years, ranging from 8 months to 13 years. The third group were 3 public health stakeholders (two government officials from the Reproductive and Child Health departments at Temeke Municipal Medical Office of Health and the Ministry of Health, Community Development, Gender, Elderly and Children (MoHCDGEC), and one mid-level hospital administrator). The characteristics of respondents are summarised in Additional file 5.

Two themes, ‘Enablers to accurate birthweight data’ and ‘Barriers to accurate birthweight data’, and eight sub-themes emerged from thematic analysis of transcripts. Reported enablers created favourable conditions for measuring and recording of quality birthweight data, while barriers created disadvantageous conditions.

### Enablers to accurate birthweight data

#### Parents and community value birthweight

Every woman described that it was necessary to weigh an infant at birth, giving nonspecific reasons for valuing birthweight as an expected component of postnatal care:

*‘What I know is that a small child should be weighed.’* - Woman, age 24 years, Temeke TZ

*‘It is important [to know the weight of my baby] so that I know where to start taking care of the baby.’* - Woman, age 36 years, Temeke TZ

Three women reported that they did, or would ask to know the birthweight, if it were not communicated to them.

One public health stakeholder described that communities knew, on a basic level, the importance of a normal birthweight:

*‘The communities understand the importance of having a baby that isn't underweight. You know, once they deliver, the first thing they ask, whether it’s the relative or the mother, "How much is the weight?" They know the importance of having a child who is a normal birthweight. They know that. Probably they are not very much aware, when the child is born underweight, what are the complications that this child is going to come to get. They know it is not good. But they do not know what has happened actually with low birthweight.’* - Public health official, age 38 years, TZ

A doctor expressed the opinion that, compared to the past, women more frequently expect that their baby be weighed after birth and express a desire to know the birthweight, although he was the only respondent to identify this trend.

#### Hospital staff value birthweight

Every healthcare provider stated that measuring birthweight was an imperative. The nurses/midwives and doctor described taking initiative after birth to find and maintain a functioning scale:

*‘A problem is that the digital weighing scales use batteries that [run out] all the time. Most of the time we try to regulate [the scales] ourselves and we buy the batteries from our own pockets. Most of the time we report [malfunctioning scales] to the management and try to bring more digital weighing machines.’* - Doctor, age 40 years, Temeke TZ

*‘We will find any means possible to weigh the baby. We cannot stop weighing the babies, how then will we make drug calculations? Weighing a baby is compulsory.’* - Nurse/midwife, age 50 years, Temeke TZ

#### Knowledge of birthweight usefulness

Women and health workers commonly stated that birthweight was an important measurement as the baseline to monitor the growth of the baby. Using birthweight to inform medication and treatment was also reported by nurses/midwives and women:

*“If a person delivers and they don’t know what the baby weighs, and the baby is sick, when they want to give you medication they will ask what the baby weighs. Therefore, I think there is as importance of knowing the weight.”* - Woman, age 22 years, Temeke TZ

Doctors and nurses/midwives knew that errors in birthweight measurement could result in dangerous administration of incorrect dosage of various medications for the infant.

A number of nurses/midwives stated that high birthweight babies could be an indicator of a health problem, such as maternal gestational diabetes, or that LBW could be a sign of poor nutrition:

*‘First and foremost a new baby has to be weighed in order to know if there is any health problem.’*- Woman, age unknown, Temeke TZ

Among women who had given birth to normal birthweight babies, the most commonly cited use of birthweight was to monitor growth. Mothers of LBW babies reported uses of birthweight were identifying health problems and informing appropriate care.

**Fig. 2:**
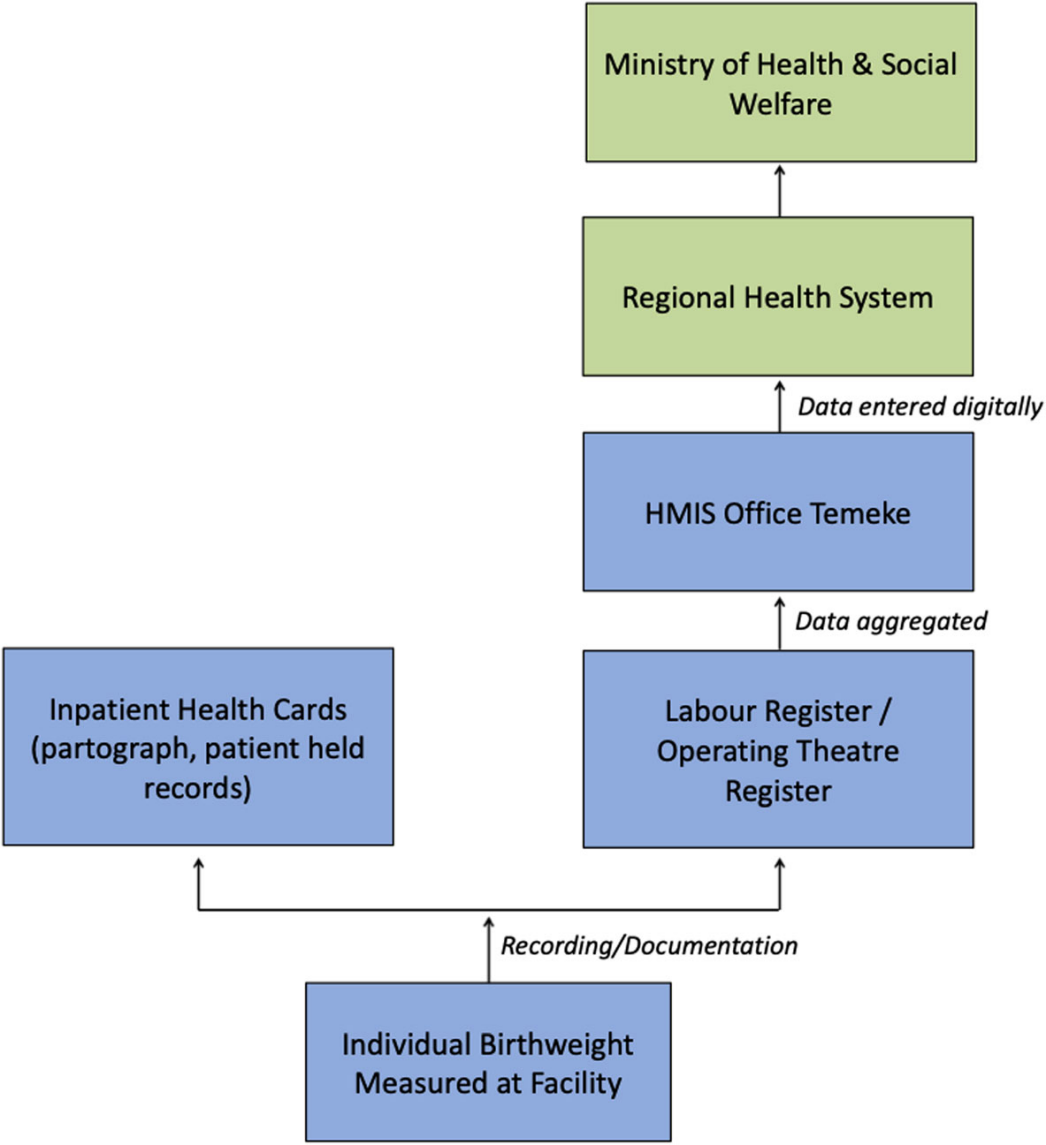
Flow of birthweight data through the digital health information system at Temeke Hospital, EN-BIRTH study

Respondents stated that birthweight data were recorded in multiple documents, including the patient case notes (partograph and patient held antenatal card), and labour ward register. Labour ward register data, aggregated by LBW and normal birthweight, is collected daily and compiled into quarterly and yearly reports that are sent from Temeke Hospital using DHIS-2 to the sub-national and national health offices (Fig. 2). These reports include summary statistics on the number of LBW babies. A public health stakeholder described that collated hospital data are monitored to observe trends in birthweight:

*‘[Birthweight trends] can give us a reflection of how much our antenatal care and interventions are working. And it can give us a call to raise an alarm that, "We are seeing more children with low birthweight, what can we do?”*- Public health stakeholder, age 38 years, TZ

### Barriers to accurate birthweight data

#### Gaps in knowledge of data utility

Despite perceiving birthweight as important, many women interviewed could not provide specific examples of how such data could be used beyond the reasons described above. The public health stakeholders agreed that women have only a general understanding about birthweight importance and attributed this to the women’s level of education. Healthcare providers doubted women’s understanding of the value of birthweight, especially if they had little education:

*‘There are mothers who are slow learners, you inform them [the birthweight] but they don’t remember it.’*- Nurse, age 50 years, Temeke TZ

Two nurses/midwives suggested that women’s value of birthweight varied depending on whether the weight was low or normal:

*‘Not many of [the women] understand. Maybe for premature babies they are very much attentive to them because they have to know if the baby is increasing [in weight] or not. For mothers with babies who have normal birthweight they don’t really understand the importance of birthweight.’* - Nurse/midwife, age 26 years, Temeke TZ

A public health official stated that nurses/midwives were not always aware of the importance of birthweight data:

*‘People [at the facilities] they don't even know. They are not motivated. This data, they don't […] know the importance of using it. They just collect information and they don't know how to take into account how this data can impact.’*- Public health stakeholder, age 38 years, TZ

#### Reported equipment gaps

A lack of sufficient and suitable weighing devices was described by every health worker and public health stakeholder as a major impediment to birthweight measurement. Although most nurses/midwives expressed that they ultimately could find a weighing scale to use, many reported that there was no scale in their ward or that it was often non-functional:

*‘Yes [a lack of scales] happens. For example, right now the batteries in the weighing machine are spent. It uses eight small batteries. Therefore, as we plan on how to buy new batteries, we don’t have a weighing machine.’*- Nurse/midwife, age unknown, Temeke TZ

Even when a scale was available, it was sometimes in poor condition. Devices were described as malfunctioning or giving imprecise measurements. Participants considered electronic scales more precise than analogue scales, however, they became inaccurate when batteries ran low. Participants also reported that it was difficult to determine the precision of their measurements as there were no other working scales to compare it to in the same ward.

*‘The weighing scale can cause inaccurate measurement. […] We do not have another machine for comparison. If it is giving us inaccurate measurement, we can never know.’*- Nurse/midwife, age 26 years, Temeke TZ

Although nurses/midwives knew of hospital technicians who could repair the scales, they stated that maintaining and repairing scales was a shared responsibility. When asked to describe the maintenance and usage of the weighing scales, no healthcare provider mentioned calibration of the scale.

#### Gaps in human resources for health

A frequently cited cause of delayed or inaccurate recorded birthweights was insufficient number of nurses/midwives to care for the growing number of births at the hospital, associated with staff exhaustion and errors in both measurement and recording:

*‘[A delay in weighing newborns] is due to insufficient staff midwives. Sometimes you might find only two staffs in the ward helping mothers to deliver babies the whole night, and one may get tired and forget to write the birthweight.’*- Nurse/midwife, age 26 years, Temeke TZ

#### Communication of birthweight to families

One doctor respondent suggested the need to improve the communication of birthweight by the nurses/midwives to the women, so that it is available for them to use as they prefer.

*‘Sometimes it is [due to] their level of education, sometimes it is [due to] their lack of exposure, but mothers are told about the weight of their babies and they forget after a very short time. They are taught but they say they don’t remember.’*- Doctor, age 40 years, Temeke TZ

#### Sub-optimal weighing practices

Nurses/midwives also explained that, if a baby’s weight was not measured at the time of birth, the newborn would be weighed at some point during the hospital stay, including weighing at discharge:

*‘If the nurse forgets to weigh the baby at the labour room, there is also a nurse who realises that for them to go home she has to weigh the baby. […] The mother has to be asked the weight of her baby, if she tells you she does not know, she has to be weighed again.’*- Nurse/midwife, age 34 years, Temeke TZ

Senior nurses/midwives reported that imprecise birthweight measurements may be due to nurses’/midwives’ weighing practices:

*‘Some of the nurses might not know how to use the weighing machines accurately. It might also happen that the nurse hasn’t balanced the weighing machine, or placed the baby without making sure that the scale is in equilibrium, thus making an error.’*- Senior nurse/midwife, age 45 years, Temeke TZ

One nurse/midwife explained that even when a more precise digital scale was available, nurses/midwives may prefer to use the less accurate manual scale that they were more familiar with.

Nurses/midwives expressed that often a baby may be weighed clothed or with an additional larger cloth (“kanga” in Swahili) on the scale to prevent the baby from getting cold and to maintain cleanliness. However, instead of zeroing the scale, nurses/midwives subtracted the approximate weight of the clothes in order to calculate a ‘true’ birthweight:

*‘In order for the weight of the baby to be accurate you have to weigh the baby when it is naked to get actual body weight. Sometimes when a baby has complications you can weigh the baby with the clothes on then you minus something like 0.5 grams. For instance, a baby might be 3.7 kilograms then we can estimate the weight to be 3.6.’*- Nurse/midwife, age 25 years, Temeke TZ

The public health stakeholders distrusted the quality of birthweight data from their localities, which included the study hospital. Although they reported monitoring trends in facility-derived birthweight data, no stakeholder could report any actions or interventions that had been informed by these trends. It was suggested that in future, birthweight data could be used to inform the creation of financial priorities or health policies surrounding LBW:

*“The fact is that the resources are somewhat limited in the country and [LBW data is] not being taken to that stage. There's no specific intervention. Maybe [the trends in LBW could] be used later on, but for the time being, it has not come out.’*- Public health official, age 38 years, TZ

## Discussion

This study is one of the first evaluations of multi-stakeholder perceptions of birthweight measurement and data. A striking finding is the high value of birthweight reported by all participants: women, health workers and public health stakeholders. Women want to know their baby’s birthweight and nurses/midwives described taking initiative to overcome logistical barriers to ensure that all newborns are weighed.

Whilst birthweight was deemed highly important, women remained unclear about the specific uses of birthweight and we found suggestions of uncertainty regarding the precision of measurements. Concerns were expressed by health workers and public health stakeholders over the value and quality of hospital birthweight data. Although our findings did not suggest a lack of value by nurses/midwives, birthweight data in Temeke Hospital showed heaping, including at 2500 g– indicative of imprecision [15, 17, 18]. We identified possible reasons for this imprecision, including suboptimal practices when measuring birthweight: e.g. subtracting the approximate weight of clothes after measuring a clothed baby, which may have contributed to rounding, digit preference or miscalculation. Though some health workers understood the importance of accurate birthweight measurement, the shortage of precise scales was perceived to be a barrier and the labour ward analogue scale was neither calibrated to zero, nor capable of weighing in 10 g increments. Delay in weighing after birth was reported to be due to nurse/midwife shortage and resulted in some babies’ ‘birthweight’ being measured and recorded at discharge instead of at birth. Newborns can lose up to 10% of their birthweight within the first few days of life, leading to further inaccuracies in true birthweight measurement if there are major delays [41]. Heaping, whereby measures are rounded, e.g. up to 2500 g, may lead to underestimation of LBW. Conversely, where birthweight measurement is delayed by a day or more, a newborn weighing over 2500 g may then weigh < 2500 g due to physiological weight loss.

Hospital birthweight data was being received regularly by the appropriate government offices and the LBW prevalence tracked, however they reported that the perceived poor quality of these data impeded its use to set priorities and inform health policies.

Given the reported high value of birthweight measurement by all respondents, opportunities exist to improve quality of hospital birthweight data. Interventions to overcome reported barriers could include: appropriate functioning, ideally digital, weighing scales at all times powered from the hospital electricity supply or with readily accessible batteries; standardised weighing protocols including clarity about removing clothes; training on precise birthweight measurement techniques.

Improving the quality of birthweight data is crucial so that the data already transmitted through HMIS to district and national-level, can be trusted to be used.

### Strengths and limitations

A strength of the study is the triangulation of findings using women’s, health workers’ and public health stakeholders’ perspectives. The qualitative results provided depth to EN-BIRTH quantitative analyses [17, 18]. Participants were offered interviews in their language of choice and saturation point was reached during interviewing of women and nurses/midwives, which lends support to the adequacy and quality of the findings. Temeke Hospital was purposively selected as an EN-BIRTH site for being a typical busy comprehensive emergency obstetric and newborn care (CEmONC) facility in Tanzania, so findings may have some generalisability transferable to other similar hospitals in Tanzania or other sub-Saharan African settings.

Limitations of the study include topics that were not specifically included in the semi-structured interview guide, such as scale calibration, and umbilical cord management (whether cut to a specific length or held up during weighing) were likely underrepresented in interviews. We included women from the KMC ward to ensure we had representation from the LBW group but acknowledge introducing selection bias as these mothers are likely to have received more specific education on birthweight/LBW, which may overrepresented birthweight knowledge. Future research could importantly assess the perceptions of pregnant women not yet exposed to birthweight practices in the facility. It was unfeasible to review results of the research with participants (‘member checks’), thus weakening the credibility of the findings.

The study was only in one CEmONC hospital in Tanzania, which limits the generalisability, and further research could explore other primary and secondary facility settings, to identify other context-specific interventions to inform improvements in coverage and quality of global birthweight data.

Implementation research is needed to understand how improved weighing protocols and practices, can sustainably enhance the quality of birthweight data, e.g. reducing heaping. Research on feasibility and efficacy of birthweight measurement training for health workers is also necessary. Further research is required to evaluate data flow in routine HMIS and if improved quality of data increases confidence in and use of birthweight data.

## Conclusion

Over the last few decades there has been a large increase in facility births [1]. Facility measured birthweight has potential to track LBW more regularly than is possible in population-based surveys [33, 42]. However, if such LBW data are to be useful, high coverage of accurate birthweights with aggregation for use in HMIS are needed. The high value of birthweight reported by women, healthcare providers and public health stakeholders in Temeke Hospital Tanzania reveals an opportunity to improve quality of birthweight measurements in order to better track LBW prevalence and drive progress towards global and national newborn and nutrition goals [43]. Future research should establish the feasibility and efficacy of interventions to improve birthweight data quality.

## Supplementary Information

**Additional file 1:** Literature review search strategy, EN-BIRTH study.

**Additional file 2:** In-depth interview guides, EN-BIRTH study.

**Additional file 3:** Qualitative coding themes.

**Additional file 4:** Consolidated criteria for reporting qualitative research (COREQ) checklist, EN-BIRTH study.

**Additional file 5:** EN-BIRTH study respondent characteristics.

**Additional file 6:** Ethical approval of local institutional review boards, EN-BIRTH study.

## Data Availability

The datasets generated during and/or analysed during the current study are available on LSHTM Data Compass repository, https://datacompass.lshtm.ac.uk/955/.

## Abbreviations

DHIS-2: District Health Information Software 2
DHS: The Demographic and Health Surveys Program
EN-BIRTH: *Every Newborn –* Birth Indicators Research Tracking in Hospitals
ENAP: *Every Newborn* Action Plan
HMIS: Health Management Information Systems
ICU: Intensive Care Unit
IHI: Ifakara Health Institute
KMC: Kangaroo Mother Care
LBW: Low Birthweight
LMIC: Low- and Middle-Income Countries
LSHTM: London School of Hygiene & Tropical Medicine
MoHCDGEC: Ministry of Health, Community Development, Gender, Elderly and Children (Tanzania)
WHO: World Health Organization
