# Supplementary material for: Birthweight measurement processes and perceived value: qualitative research in one EN-BIRTH study hospital in Tanzania
Source: BMC Pregnancy Childbirth. 2021 Mar 26;21(Suppl 1):232. doi: 10.1186/s12884-020-03356-2 (PMC7995566; doi:10.1186/s12884-020-03356-2)
Supplement: Supplementary file 1 — Additional file 1: Literature review search strategy, EN-BIRTH study. [file 12884_2020_3356_MOESM1_ESM.pdf]

SUPPLEMENT TITLE:
Every Newborn BIRTH multi-country validation study: informing measurement of coverage and quality of maternal and newborn care

PAPER TITLE:
Birthweight measurement processes and perceived value: qualitative research in one EN-BIRTH study hospital in Tanzania

Additional File 1: Literature review search strategy, EN-BIRTH study

Refining the Question

Clear, answerable questions were developed through a review of key research papers, provided by the LSHTM team, and consultation with advisors at LSHTM, and Temeke. The overarching research questions were:

- What is the perceived value of accurate facility birthweight measurement and recording in Tanzania?
- What do initial quantitative data on birthweight reveal about the measurement and recording practices?

Specific research questions included:

- What data exists in literature on the perceptions of the importance of birthweight measurement and recording in facility births?
- How does the valuation of birthweight influence the collection and recording of birthweight?
- What is the nature of collected birthweight data in regard to quality and completeness?
- How is birthweight data used by hospitals and the Ministry of Health to decrease maternal and neonatal mortality?

Choosing the Database

The Global Health database was accessed, through the Ovid interface, due to its abundance of peer-reviewed papers on international public health.

Search Terms

Search terms were created from the research questions and were mapped to their subject headings. A search strategy was assembled using Boolean operators to combine the search terms. The search strategy was:

birth weight/ or low birth weight infants/ AND beliefs/ AND exp attitudes/ or exp public opinion/

The search yielded four relevant papers.

Expanding the Search

The reference sections of the papers found in the database and those provided by the ENAP team were examined and used to identify other relevant literature.
