# Supplementary material for: Birthweight measurement processes and perceived value: qualitative research in one EN-BIRTH study hospital in Tanzania
Source: BMC Pregnancy Childbirth. 2021 Mar 26;21(Suppl 1):232. doi: 10.1186/s12884-020-03356-2 (PMC7995566; doi:10.1186/s12884-020-03356-2)
Supplement: Supplementary file 2 — Additional file 2: In-depth interview guides, EN-BIRTH study. [file 12884_2020_3356_MOESM2_ESM.pdf]

SUPPLEMENT TITLE:
Every Newborn BIRTH multi-country validation study: informing measurement of coverage and quality of maternal and newborn care

PAPER TITLE:
Birthweight measurement processes and perceived value: qualitative research in one EN-BIRTH study hospital in Tanzania

Additional File 2: In-depth interview guides, EN-BIRTH study

In-depth interview guide for women

Introduction:

Before we begin, I want to thank you for agreeing to talk to me today. My name is __________ and I would like to talk to you to about your experiences giving birth in Temeke Regional Referral Hospital. Specifically, we are interested in your thoughts on your baby's birthweight to. This information will help us to improve the birthweight measurement and recording practices in this hospital.

This interview should take about thirty minutes. I will be recording the interview so that I don't miss any of your comments. I will also be taking some notes during our discussion.

Everything you say will be kept confidential. You have been assigned a number so that your responses are never linked to your name. What you say will only be shared with the research team and will never identify you. You don't have to answer any questions you don't want to and you may end the interview any time you wish.

Do you have any questions about what I just explained? Can you please take a moment to review the information and consent form? Please let me know if you need a question to be clarified.

Background

1. How many times have you given birth in your life?
2. How many times have you given birth at this hospital?
3. How many babies have you had this time?
4. Did you attend antenatal care? If so, where?

Birthweight Recording Practice:

Note to interviewer – in the case of a multiple birth, prompt the mother to answer for both babies

5. Was your baby weighed after birth?
    - If not, do you know why not?
    - If so, did you see the baby weighed? What did you see?

- Do you know what type of scale was used?
- Do you know how long after birth the baby was weighed?
- What was the baby wearing when it was weighed? (Clothing or medical devices)

6. Were you told how much your baby weighed?
   - If so, who told you? When were you told?
   - If not, did you ask how much he/she weighed?
7. How much did your baby weigh?
8. Did you record the weight anywhere? If so, where?

Perceptions of Accurate Birthweight Measurement

9. Why do you think your baby was/wasn't weighed at birth?
10. Was it important to you that you knew your baby's weight? Why or why not?
11. Do you think it is important to weigh babies when they are born? Why or why not?
12. Did anyone explain to you the importance of your baby's weight?
13. If applicable, do you remember the weight of your other babies at birth? If so, what were they?

Closing Remarks:

We have reached the end of the interview, but before we finish is there anything else you would like to add?

I'll be analysing the information that you and others have given me and I will be submitting a report during September. I will send a copy to the hospital and you are welcome to review it at that time if you are interested.

Thank you for your time.

In-depth interview guide for nurses

Introduction

Thank you for taking the time to participate in this focus group discussion. My name is ________ and I am from the London School of Hygiene and Tropical Medicine researching attitudes towards birthweight measurement and recording. You have been invited to participate in this discussion because of your experience with measuring and recording birthweight in Temeke Hospital.

Everything you say during this discussion will be kept strictly confidential. You have been assigned a number that will ensure that what you say is never associated with your name. You do not have to participate in this interview if you don't want to and you don't have to talk about anything you don't want to. I have an information sheet that explains what I've just told you. Please take a moment to read it. Do you have any questions about the information on this sheet?

I will be recording this conversation and taking notes so that we can remember what you tell us. We will destroy the recording after the discussion has been transcribed. Could you please read this consent form? Do you have any questions about the consent form? If you consent to participate in this discussion can you please sign the consent form.

Background:

1. Can you tell me what your role is in the health facility? How long have you each been working in your current role?
2. What are your responsibilities during a birth?
3. What is your age?

Birthweight Measurement & Recording Practices:

4. What steps do you take to care for the baby immediately after it is born?
5. Can you describe how you weigh babies?

Note to interviewer: If the information isn't provided, prompt for information on type of scale, timing of weighing baby, whether the baby is dressed/has medical devises attached etc.

6. What type of weighing devices do you have in your facility? Who is responsible for caring for the scales?
7. Have you ever had a problem with not having a working scale to weigh a baby on? How did you solve the problem?
8. Can you think reasons that:

    - a baby would not be weighed?
    - a baby would not be naked when weighed?
    - the recorded birthweight would be inaccurate?

Birthweight Data:

9. Where is the birthweight recorded after it is measured?
10. Do you inform mothers of their baby's birthweight? Why or why not?
11. How is the mother informed of her baby's birthweight?

Perceptions of Accurate Birthweight Measurement:

12. Have you always done things as you have described or has there been any change in your practises over the years?
    - If there have been changes, what were the reasons for these changes?
13. Do you think it is important to measure birthweight? Why or why not?
14. What does a baby's birthweight tell you about the baby? Does knowing the baby's birthweight change how you care for the baby?

Closing Remarks:

We have reached the end of the discussion, but before we finish is there anything else you would like to add?

I'll be analysing the information that you and others have given me and I will be submitting a report during September. I will send a copy to the hospital and you are welcome to review it at that time if you are interested.

Thank you for your time.

In-depth interview guide for hospital stakeholders

Introduction:

Thank you for taking the time to participate in this interview. My name is ____________ and I from the London School of Hygiene and Tropical Medicine researching attitudes towards birthweight measurement and recording. You have been invited to participate in this interview because of your experience at Temeke Hospital.

Everything you say during this discussion will be kept strictly confidential. You have been assigned a number that will ensure that what you say is never associated with your name. You do not have to answer any question you don't want to. I have an information sheet that explains what I've just told you. Please take a moment to read it. Do you have any questions about the information on this sheet?

I will be recording this conversation and taking notes so that we can remember what you tell us. We will destroy the recording after the interview has been transcribed. Could you please read this consent form? Do you have any questions about the consent form? If you consent to participate in this discussion can you please sign the form? Please let me know if you need a question to be clarified.

Background:

1. What is your role at the hospital?
2. How long have you been in this role?
3. What is your age?

Birthweight Measurement & Recording Practices:

4. What type of weighing devices do you have in your facility? Who is responsible for taking care of these weighing devices?
5. What is the procedure for weighing a baby after it is born?
6. Can you think of reasons that:

   - A baby would not be weighed?
   - A baby would not be naked when weighed?
   - The recorded birthweight would be inaccurate?

Birthweight Data:

7. Where is the birthweight recorded after it is measured?
8. When do mothers find out the birthweight of their child?
9. Do you use birthweight data in your day-to-day job?
    - If so, can you tell me a bit about how and when you use birthweight data?

Perceptions of Accurate Birthweight Data

10. Why do you think measuring birthweight is or is not important?
11. In your opinion, what are areas requiring improvement in birthweight collection and recording in your ward?
12. What do you think are the greatest barriers to accurate birthweight measurement and recording in your ward?
13. In your opinion, what are areas that have been successful in birthweight measurement in your ward?
14. What is the greatest enabler of accurate birthweight measurement and recording?
15. Do you think that mothers understand the significance of their infant's birthweight?
16. Do you think there have been any changes in the measurement and recording of birthweight data in your ward while you've been in this job?

Closing Remarks:

We have reached the end of the interview, but before we finish is there anything else you would like to add?

I'll be analysing the information that you and others have given me and I will be submitting a report during September. If you are interested, I will send a copy to you and you are welcome to review it at that time.

Thank you for your time.

In-Depth interview guide for MOHSW/Municipal stakeholders

Introduction:

Thank you for taking the time to participate in this interview. My name is __________ and I am from the London School of Hygiene and Tropical Medicine researching attitudes towards birthweight measurement and recording.

Everything you say during this interview will be kept strictly confidential. You have been assigned a number that will ensure that what you say is never associated with your name. You do not have to answer any questions you don't want to and you don't have to talk about anything you don't want to. I have an information sheet that explains what I've just told you. Please take a moment to read it. Do you have any questions about the information on this sheet?

I will be recording this conversation and taking notes so that we can remember what you tell us. We will destroy the recording after the discussion has been transcribed. Could you please read this consent form? Do you have any questions about the consent form? If you consent to participate in this discussion can you please sign the form? Please let me know if you need a question to be clarified.

Background:

1. What is your role in the Ministry of Health?
2. How long have you been in this role?
3. What is your age?

Data Flow of Birthweight:

6. What happens to birthweight data after it is collected in the health facility?
7. Do you use birthweight data in your day-to-day job?
    - If so, can you tell me a bit about how and when you use birthweight data? 6. Does collected birthweight data impact health programming? How?

Perceptions of Accurate Birthweight Data

8. Why do you think measuring birthweight is important or not important?
9. In your opinion, what are areas requiring improvement in birthweight data collection in Tanzania?
10. In your opinion, what are areas that have been successful in birthweight data collection in Tanzania?
11. Do you think that mothers understand the significance of their infant's birthweight?
12. How do you think that inaccurate birthweight data would impact maternal and newborn health?
13. Do you think there have been any changes in relation to the measurement and recording of birthweight data in Tanzania while you have been working at the Ministry of Health?

Closing Remarks:

We have reached the end of the interview, but before we finish is there anything else you would like to add?

I'll be analysing the information that you and others have given me and I will be submitting a report during September. If you are interested, I will send a copy to you and you are welcome to review it at that time.

Thank you for your time.
