# Supplementary material for: Birthweight measurement processes and perceived value: qualitative research in one EN-BIRTH study hospital in Tanzania
Source: BMC Pregnancy Childbirth. 2021 Mar 26;21(Suppl 1):232. doi: 10.1186/s12884-020-03356-2 (PMC7995566; doi:10.1186/s12884-020-03356-2)
Supplement: Supplementary file 3 — Additional file 3: Qualitative coding themes. [file 12884_2020_3356_MOESM3_ESM.pdf]

SUPPLEMENT TITLE:
Every Newborn BIRTH multi-country validation study: informing measurement of coverage and quality of maternal and newborn care

PAPER TITLE:
Birthweight measurement processes and perceived value: qualitative research in one EN-BIRTH study hospital in Tanzania

Additional File 3: Qualitative coding themes, EN-BIRTH study

Perceived Barriers to Accurate Birthweight Data

a. *Attitudes and Knowledge*
    i. Community Lack of Knowledge on Weight
        - Survival of LBW babies
    ii. Mother's Understanding of Weight
        - Lack of Knowledge on Weight
        - Mother's Emotional State
        - Mother's Education Level
    iii. Staff Knowledge and Attitudes
        - Fragility of Baby (e.g. Maintaining cleanliness and preventing cold)
        - Lack of Knowledge (e.g. On operating scales, using data)

b. *Resources*
    i. Human Resources
        - Exhaustion of Nurses
        - Increase in Number of Deliveries
        - More Patients Than Staff
    ii. Physical Resources
        - Availability of Scale
        - Condition of Scale (e.g. Batteries, maintenance)

c. *Practices*
    i. Mother's Actions
        - Observations of Weighing
        - Recall of Weighing
    ii. Nurses' Actions
        - Delay in Weighing (e.g. Due to complications, or forgetting to weigh)
    iii. Stakeholders' Actions
        - Poor usage of birthweight data on population level

Perceived Facilitators to Accurate Birthweight Data

a. Attitudes of Importance of Birthweight
    i. Mother's Understanding
        - Improvement in maternal comprehension
        - Education of mothers (e.g. Antenatal and hospital education)

        - Fundamental comprehension of weight (e.g. Emotional response, asking to know weight)
    ii. Staff's Understanding
        - Weighing newborns is always necessary (e.g. Due to routine, expectation, or mother's right to know)

b. Known Uses of Birthweight
    i. Individual Level
        - Track progress of growth
        - Identify Health Problems (e.g. Gestational diabetes, poor nutrition)
        - Inform Appropriate Care (e.g. Feeding, medication, KMC)
    ii. Population Level
        - Monitoring birthweight trends (e.g. Identifying population health issues, changes in prevalence of LBW)

c. Practices
    i. Mother's Actions
        - Retaining antenatal card
        - Asking to know weight
    ii. Staff's Actions
        - Taking initiative to increase likelihood of baby being weighed (e.g. Double checking weights, weighing at discharge)
