# Supplementary material for: Birthweight measurement processes and perceived value: qualitative research in one EN-BIRTH study hospital in Tanzania
Source: BMC Pregnancy Childbirth. 2021 Mar 26;21(Suppl 1):232. doi: 10.1186/s12884-020-03356-2 (PMC7995566; doi:10.1186/s12884-020-03356-2)
Supplement: Supplementary file 4 — Additional file 4: Consolidated criteria for reporting qualitative research (COREQ) checklist, EN-BIRTH study. [file 12884_2020_3356_MOESM4_ESM.pdf]

SUPPLEMENT TITLE:
Every Newborn BIRTH multi-country validation study: informing measurement of coverage and quality of maternal and newborn care

PAPER TITLE:
Birthweight measurement processes and perceived value: qualitative research in one EN-BIRTH study hospital in Tanzania

Additional File 4: Consolidated criteria for reporting qualitative research (COREQ) checklist, EN-BIRTH study

	Item No	Guide questions/description	Reported Y/N
**Research team and reflexivity**			
Personal Characteristics			
Interviewer/facilitator	1	Which author/s conducted the interview or focus group?	Y
Credentials		What were the researcher's credentials? E.g. PhD, MD	Y
Occupation	3	What was their occupation at the time of the study?	Y
Gender	4	Was the researcher male or female?	Y
Experience and training	5	What experience or training did the researcher have?	Y
Relationship with participants			
Relationship established	6	Was a relationship established prior to study commencement?	Y<br>.
Participant knowledge of the interviewer	7	What did the participants know about the researcher? e.g. personal goals, reasons for doing the research	In-Depth Interview Guides (Additional Files)
Interviewer characteristics	8	What characteristics were reported about the inter viewer/facilitator? e.g. Bias, assumptions, reasons and interests in the research topic	In-Depth Interview Guides (Additional Files)

**Domain 2: study design**			
Theoretical framework			
Methodological orientation and Theory	9	What methodological orientation was stated to underpin the study? e.g. grounded theory, discourse analysis, ethnography, phenomenology, content analysis	Y
Participant selection			
Sampling	10	How were participants selected? e.g. purposive, convenience, consecutive, snowball	Y
Method of approach	11	How were participants approached? e.g. face-to-face, telephone, mail, email	Y
Sample size	12	How many participants were in the study?	Y
Non-participation	13	How many people refused to participate or dropped out? Reasons?	Y
Setting			
Setting of data collection	14	Where was the data collected? e.g. home, clinic, workplace	Y .
Presence of non-participants	15	Was anyone else present besides the participants and researchers?	Y
Description of sample	16	What are the important characteristics of the sample? e.g. demographic data, date	Y
Data collection			
Interview guide	17	Were questions, prompts, guides provided by the authors? Was it pilot tested?	Y
Repeat interviews	18	Were repeat interviews carried out? If yes, how many?	Y
Audio/visual recording	19	Did the research use audio or visual recording to collect the data?	Y
Field notes	20	Were field notes made during and/or after the interview or focus group?	Y
Duration	21	What was the duration of the inter views	Y

		or focus group?	
Data saturation	22	Was data saturation discussed?	Y
Transcripts returned	23	Were transcripts returned to participants for comment and/or correction?	Y
**Domain 3: analysis and findings**			
Data analysis			
Number of data coders	24	How many data coders coded the data?	Y
Description of the coding tree	25	Did authors provide a description of the coding tree?	Y
Derivation of themes	26	Were themes identified in advance or derived from the data?	Y
Software	27	What software, if applicable, was used to manage the data?	Y
Participant checking	28	Did participants provide feedback on the findings?	Y
Reporting			
Quotations presented	29	Were participant quotations presented to illustrate the themes/findings? Was each quotation identified? e.g. participant number	Y
Data and findings consistent	30	Was there consistency between the data presented and the findings?	Y
Clarity of major themes	31	Were major themes clearly presented in the findings?	Y
Clarity of minor themes	32	Is there a description of diverse cases or discussion of minor themes?	Y
