# Supplementary material for: Birthweight measurement processes and perceived value: qualitative research in one EN-BIRTH study hospital in Tanzania
Source: BMC Pregnancy Childbirth. 2021 Mar 26;21(Suppl 1):232. doi: 10.1186/s12884-020-03356-2 (PMC7995566; doi:10.1186/s12884-020-03356-2)
Supplement: Supplementary file 5 — Additional file 5: EN-BIRTH study respondent characteristics. [file 12884_2020_3356_MOESM5_ESM.pdf]

SUPPLEMENT TITLE:
Every Newborn BIRTH multi-country validation study: informing measurement of coverage and quality of maternal and newborn care

PAPER TITLE:
Birthweight measurement processes and perceived value: qualitative research in one EN-BIRTH study hospital in Tanzania

Additional File 5: EN-BIRTH study respondent characteristics

Table of Characteristics of Respondents (Women)

Type of Respondent	N	Age				Parity			Education Level			
		19-30	30-40	>40	Unknown	1	2-4	>4	None	Primary	Secondary	Unknown
Woman with baby of normal weight	4	4	-	-		1	3	-	-	-	4	
Woman with LBW baby	4	1	2	-	1	1	1	2	1	2	-	1

Table of Characteristics of Respondents (Other)											
Type of Respondent	*N*	Age					Sex		Length of Employment at Current Employer		
		<19	*19-30*	*30-40*	*>40*	*Unknown*	*Male*	*Female*	*< 3 years*	*3-5 years*	*>5 years*
Health Care Provider	10	-	2	1	6	1	2	8	4	3	3
Public Health Stakeholder	3	-	-	2	-	1	1	2	-	2	1
